# Supplementary material for: Monoallelic TYROBP deletion is a novel risk factor for Alzheimer’s disease
Source: Mol Neurodegener. 2025 Apr 29;20:50. doi: 10.1186/s13024-025-00830-3 (PMC12038944; doi:10.1186/s13024-025-00830-3)
Supplement: Supplementary file 1 — Additional file 1 [file 13024_2025_830_MOESM1_ESM.docx]

Monoallelic *TYROBP* deletion is a novel risk factor for Alzheimer’s disease

Henna Martiskainen^1#^*, Roosa-Maria Willman^1#^, Päivi Harju^1^, Sami Heikkinen^1^, Mette Heiskanen^1^, Stephan A. Müller^2,3^, Rosa Sinisalo^1^, Mari Takalo^1^, Petra Mäkinen^1^, Teemu Kuulasmaa^1^, Viivi Pekkala^1,4^, Ana Galván del Rey^1^, Sini-Pauliina Juopperi^1^, Heli Jeskanen^1^, Inka Kervinen^1^, Kirsi Saastamoinen^1^, FinnGen†, Marja Niiranen^5^, Sami V. Heikkinen^6^, Mitja I. Kurki^7,8,9,10^, Jarkko Marttila^11^, Petri I. Mäkinen^4^, Hannah Rostalski^4,12,13^, Tomi Hietanen^4^, Tiia Ngandu^,14,15^, Jenni Lehtisalo^6,14^, Céline Bellenguez^16^, Jean-Charles Lambert^,16^, Christian Haass^2,17,18^, Juha Rinne ^19,20^, Juhana Hakumäki^11,21^, Tuomas Rauramaa ^22,23^, Johanna Krüger ^24,25,26^, Hilkka Soininen^6^, Annakaisa Haapasalo^4^, Stefan F. Lichtenthaler^2,3,18^, Ville Leinonen ^27,28^, Eino Solje^28,29^, and Mikko Hiltunen^1^*

^1^Institute of Biomedicine, University of Eastern Finland; Kuopio, Finland.

^2^German Center for Neurodegenerative Diseases (DZNE); Munich, Germany.

^3^Neuroproteomics, School of Medicine and Health, Technical University of Munich; Munich, Germany.

^4^A. I. Virtanen Institute for Molecular Sciences, University of Eastern Finland; Kuopio, Finland.

^5^Neuro Center - Neurology, Kuopio University Hospital; Kuopio, Finland.

^6^Institute of Clinical Medicine - Neurology, University of Eastern Finland; Kuopio, Finland.

^7^Institute for Molecular Medicine Finland (FIMM), Helsinki Institute of Life Science (HiLIFE), University of Helsinki; Helsinki, Finland.

^8^Program in Medical and Population Genetics, Broad Institute of Harvard and MIT; Cambridge, MA, USA.

^9^Stanley Center for Psychiatric Research, Broad Institute of Harvard and MIT; Cambridge, MA, USA.

^10^Analytic and Translational Genetics Unit, Massachusetts General Hospital; Boston, MA, USA.

^11^Department of Clinical Radiology, Imaging Center, Kuopio University Hospital; Kuopio, Finland.

^12^Department of Clinical Medicine and Biotech Research and Innovation Centre (BRIC), University of Copenhagen, Copenhagen, Denmark^13^The Bartholin Institute, Department of Pathology, Rigshospitalet, Copenhagen University Hospital, Copenhagen, Denmark

^14^Department of Public Health, Finnish Institute for Health and Welfare, Helsinki, Finland.

^15^Division of Clinical Geriatrics, Center for Alzheimer Research, Department of Neurobiology, Care Sciences and Society, Karolinska Institutet, Stockholm, Sweden.

^16^Université de Lille, Inserm, CHU Lille, Institut Pasteur de Lille, LabEx DISTALZ - U1167-RID-AGE Facteurs de risque et déterminants moléculaires des maladies liées au vieillissement; Lille, France.

^17^Metabolic Biochemistry, Biomedical Centre (BMC), Faculty of Medicine, Ludwig-Maximilian University of Munich; Munich, Germany.

^18^Munich Cluster for Systems Neurology (Synergy); Munich, Germany.

^19^Turku PET Centre, Turku University Hospital; Turku, Finland.

^20^ InFLAMES Research Flagship Center, University of Turku; Turku, Finland.

^21^ Unit of Radiology, Institute of Clinical Medicine, University of Eastern Finland; Kuopio, Finland.

^22^Department of Clinical Pathology, Kuopio University Hospital; Kuopio, Finland.

^23^Unit of Pathology, Institute of Clinical Medicine, University of Eastern Finland; Kuopio, Finland.

^24^Research Unit of Clinical Medicine, Neurology, University of Oulu; Oulu, Finland.

^25^Medical Research Center, Oulu University Hospital; Oulu, Finland.

^26^Neurocenter, Neurology, Oulu University Hospital; Oulu, Finland.

^27^Department of Neurosurgery, Kuopio University Hospital; Kuopio, Finland.

^28^Institute of Clinical Medicine, University of Eastern Finland; Kuopio, Finland.

^29^Neuro Center - Neurology, Kuopio University Hospital; Kuopio, Finland.

Supplementary Materials and Methods

Supplementary Figures 1-7

Supplementary Materials and Methods

**Immunocytochemistry**

For immunocytochemistry, MDMi cells and monocytes were cultured in 96-well Ibidi µ-slides (#89626, Ibidi, Mediq, Finland) at a density of 90,000 cells per well. Monocytes, isolated from PBMCs as previously described, were kept for 24 hours in RPMI-1640 Glutamax (61870010, Gibco) medium supplemented with 10% FBS (A5256801, Thermo Fisher) and 1% penicillin/streptomycin (ECB3001D, BioNordica, Finland). MDMi were differentiated from monocytes until DIV 11 as described above.

Cells were first fixed with 4% PFA (28908, Thermo Fisher) for 10 minutes and washed twice with PBS. For intracellular staining, cells were permeabilized with 0.1% Triton X-100 (BDH Laboratory Supplies, England) for 10 minutes, followed by a single PBS wash. Blocking was performed using 5% BSA (A9647-100G, Sigma-Aldrich) in PBS for 30 minutes at RT, after which the cells were incubated overnight at 4°C with primary antibodies diluted in 1% BSA/PBS. The primary antibodies were anti-CX3CR1 (702321, Thermo Fisher, 1:200), anti-IBA1 (019-19741, Wako, 1:400), anti-P2RY12 (HPA014518, Sigma-Aldrich, 1:125), anti-PU.1 (2266S, Cell Signaling Technology, 1:200), anti-TMEM119 (HPA051870, Thermo Fisher, 1:250) and anti-TREM2 (91068S, Cell Signaling Technology, 1:200). Negative control wells without primary antibodies were processed simultaneously.

Following overnight incubation, the cells were washed three times with PBS and incubated for 1 h at RT in the dark with an Alexa488-conjugated secondary antibody (A-21206, Thermo Fisher, 1:500) diluted in 1% BSA/PBS. After three additional PBS washes, nuclei were stained with DAPI (D1306, Thermo Fisher, 1 mg/ml, 1:1,000) for 5 min at RT in the dark. Finally, the cells were washed three times with PBS and stored in PBS at 4°C, protected from light. Images were acquired using a Zeiss LSM700 confocal laser scanning microscope (Carl Zeiss, Germany) with a 20x objective. No unspecific staining was observed in the negative control samples. Exposure settings were kept constant for each primary antibody. Image processing was performed using ImageJ (1.54g, NIH, USA).

**Creation and validation of *Tyrobp* KO BV2 cell lines**

Immortalized mouse microglial BV2 cells were cultured in RPMI-1640 medium (R0883-500ML, Sigma-Aldrich) supplemented with 10 % fetal bovine serum (10500064, Thermo Fisher), 1 % penicillin-streptomycin (DE17-602E, Lonza™), and 2 mM L-glutamine (BE17-605E, Thermo Fisher) in humidified atmosphere in 37ºC with 5 % CO2. For the experiments, the concentration of FBS was lowered to 5%.

To generate *Tyrobp* KO cell lines, BV2 cells were transduced with LV01 all-in-one CRISPR-Cas9 lentiviral vector targeting exon three of *Tyrobp* (MMPD0000041569, Sigma-Aldrich). To generate control lines, LV01 non-targeting NegativeControl1 vector was used.

A total of 600,000 cells were seeded on 6-well plates and let to adhere for 2 h before adding the viral vector at multiplicity of infection 10 together with 8 µg/ml of polybrene (TR-1003-G, Sigma-Aldrich). After 21 hours, the virus containing medium was replaced with fresh medium, and the puromycin selection (3 µg/ml, P8833-10MG, Sigma-Aldrich) was started four hours later. After four days of selection, monoclonal lines were established by sorting single GFP-positive cells on 96-wells by fluorescence-activated cell sorting. Monoclonal cell populations were expanded, and DNA and protein samples were collected for determining the knock-out lines.

Sanger sequencing for *Tyrobp* exon three was performed to identify clones with successful gene editing. The region was amplified by PCR using primers 5’-TTCTCCTTAGGATTAAGTCCCGT-3’ (forward) and 5’-TGTGACCTTGACGCTTCCAC-3’ (reverse), and the product was purified with ExoSAP-IT™ PCR Product Cleanup Reagent (78201.1.ML, Thermo Fisher Scientific). The sequencing reaction was performed using BigDye™ Terminator v3.1 kit (4337455, Thermo Fisher Scientific), and after ethanol/EDTA precipitation, the pure product was resuspended in Hi-Di™ Formamide (4311320, Thermo Fisher Scientific). The sequencing was performed at Genome Center of Eastern Finland. TIDE online tool (http://shinyapps.datacurators.nl/tide/) was used to investigate the generated indels in the lines.

**siRNA transfection**

BV2 cells were transfected with siRNA targeting mouse *Tyrobp* (s75642, Invitrogen), or non-targeting negative control siRNA (4390843, Invitrogen) using Lipofectamine RNAiMAX (13778075, Invitrogen) according to the manufacturer’s instructions. Briefly, siRNA and Lipofectamine were diluted in Opti-MEM reduced serum medium (31985062, Gibco), combined, and incubated for 5 min at room temperature. 25 pmol of siRNA per 12 million cells was used, and the siRNA-Lipofectamine mixture was plated together with the cells on 150 mm dish and incubated for 20 h. Fresh medium was added to the cells two hours before starting the starvation.

**M-CSF treatment**

BV2 cells transfected with *Tyrobp* or non-targeting control siRNA were placed in RPMI-1640 medium without FBS to induce serum starvation for 2 h. Next, the cells were treated with 100 ng/ml recombinant human M-CSF (BioLegend cat. 574,806, San Diego, CA, USA) or PBS as vehicle control for 5 min. Samples were then placed on ice and centrifugated at 10,000 x *g* for 1 min at 4°C. Supernatant was removed, and the cell pellet was lysed in RIPA supplemented with protease and phosphatase inhibitors (Halt™ 78420 and 87786, Thermo Fisher), mixed, and incubated on ice for 10 min. Samples were centrifugated at 16,000 x g for 10 min at 4°C, and the supernatants were stored at -20°C.

**Western immunoblotting**

Western immunoblotting was carried out as previously (26). Proteins were detected using the following primary antibodies diluted in the appropriate ratio with 1x TBST: Recombinant rabbit anti-mouse DAP12 [EPR24244-119] (1:1,000, ab280568, Abcam), mouse anti-β-actin (1:1,000, ab8226, Abcam), rabbit anti-phospho-Syk Tyr525/526 (1:1,000, #2711, Cell Signaling Technology), and rabbit anti-Syk [D3Z1E] (1:1000, #13198, Cell Signaling Technology). HRP-conjugated secondary antibodies used were anti-rabbit-HRP (1:5,000 NA934 Cytiva), and sheep anti-mouse-HRP (1:5,000, NA931V, GE Healthcare, Chicago, IL, USA). Two-way ANOVA followed by Tukey’s post hoc test was used to analyze Western blot data from BV2 cells.


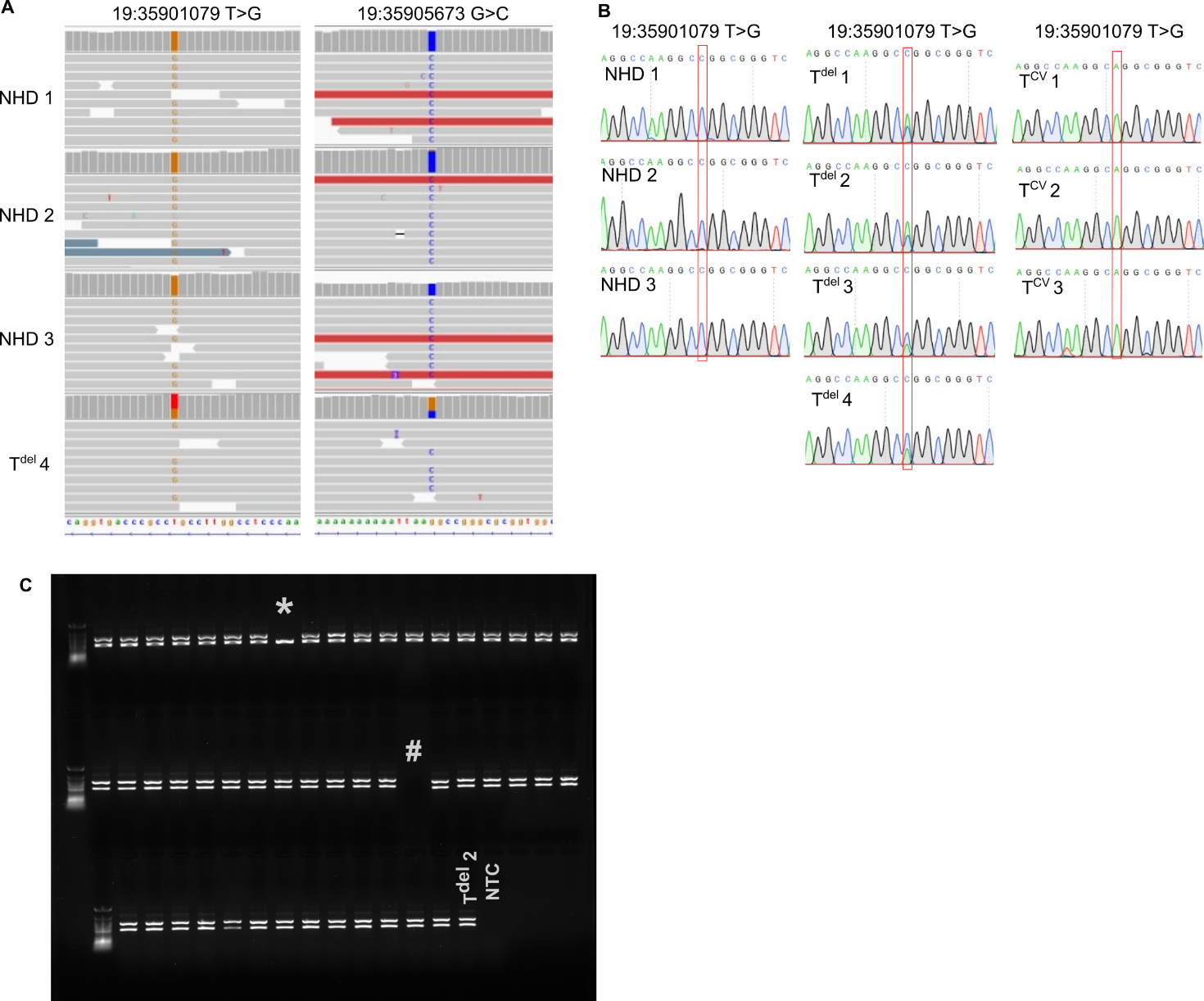


**Fig. S1 Validation of the 5.2-kb *TYROBP* deletion proxy markers.** (A) 5.2-kb *TYROBP* deletion proxy markers identified in the whole genome sequencing data of three NHD patients (homozygous for the 5.2-kb *TYROBP* deletion) and one monoallelic deletion carrier. (B) *TYROBP* deletion proxy marker rs1244787406 (19:35901079 T>G) was confirmed with Sanger sequencing in three NHD patients, three monoallelic *TYROBP* deletion carriers, and three individuals with the common variant of *TYROBP*. (C) Deletion-specific PCR of 50 imputed 19:35901079 T>G carriers from the FinnGen cohort. Two bands (~500 kb and ~700 kb) indicate individuals heterozygous for the *TYROBP* deletion, while single 500-kb band indicates an individual homozygous for the common variant of *TYROBP*. * denotes an individual with low (0.52) genotype probability for the 19:35901079 T>G variant. # denotes an empty well. NHD, Nasu Hakola disease patient; NTC, non-template control; T^del^, monoallelic *TYROBP* deletion carrier; T^CV^, *TYROBP* common variant.


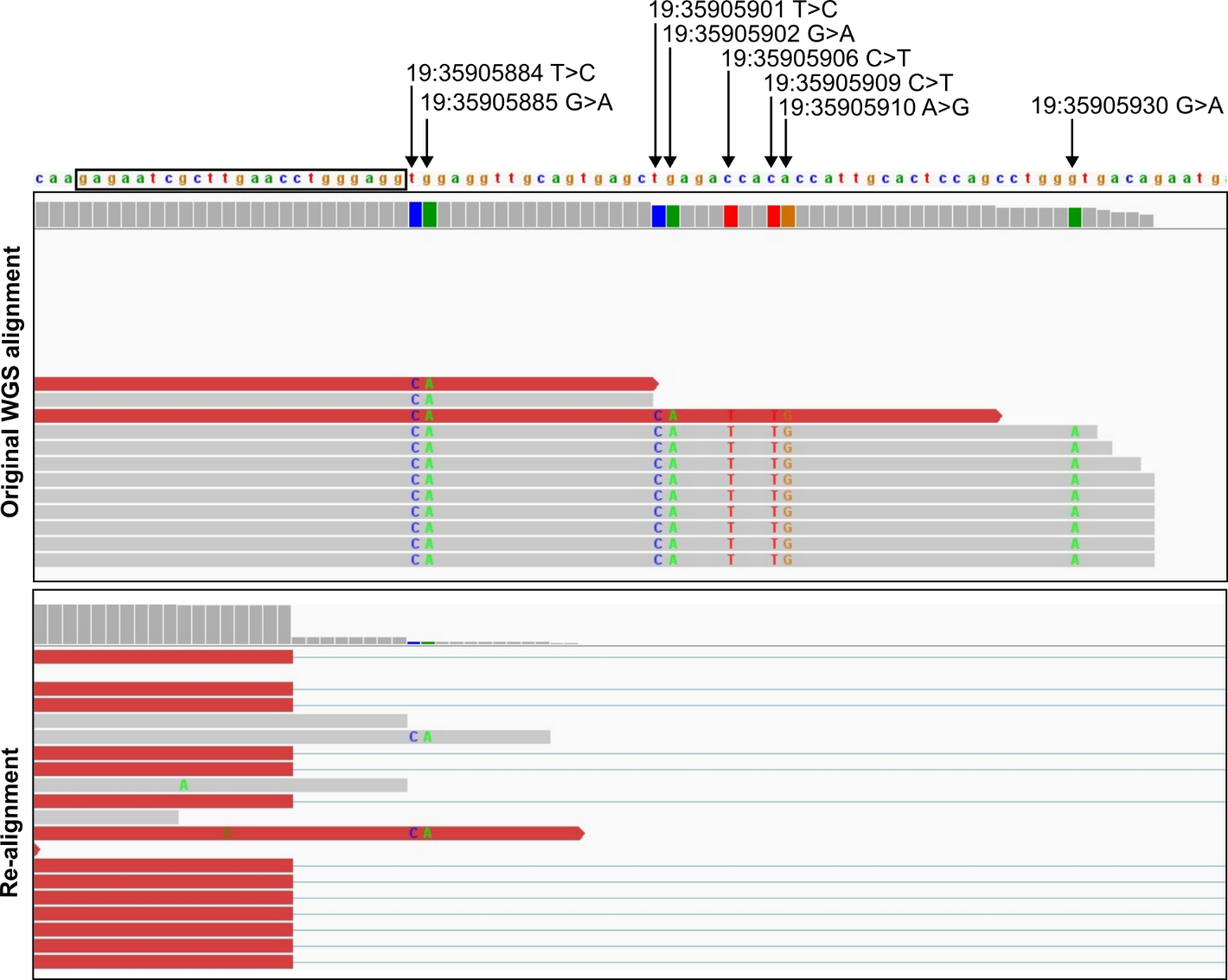


**Fig. S2 Artefact single nucleotide variants arise from incorrect alignment of WGS reads spanning the 5.2-kb *TYROBP* deletion.** The upper panel shows the original alignment of WGS data from an NHD patient with homozygous 5.2-kb *TYROBP* deletion. The lower panel shows the same data after re-alignment with parameters allowing large deletions. Horizontal lines in the lower panel indicate reads that span across the deletion. The deletion 5’ and 3’ break points are located within a 23-bp identical sequence (indicated in the figure with a black box) within 120 bp almost identical *Alu* repeats. This sequence similarity leads to incorrect alignment of the reads that span the deletion when standard parameters for WGS read alignment are used and to the detection of artefact SNVs at chromosome positions within the deleted region (indicated by arrows and chromosomal location in the figure).


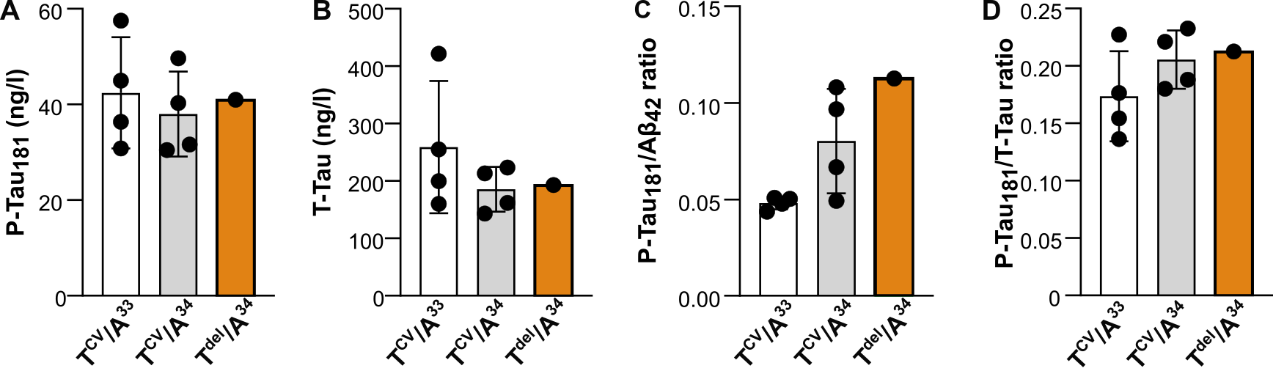


**Fig. S3 Effect of the Finnish 5.2-kb *TYROBP* deletion on AD-related Tau pathology in the CSF**. A) P-Tau181, B) total Tau (T-Tau), C) P-Tau181/Aβ42 ratio, and D) P-Tau181/T-Tau ratio in the CSF is in the same range for the monoallelic *TYROBP* deletion carrier and individuals without the *TYROBP* deletion. n=1-4 individuals per genotype. Data are shown as mean ± SD. Independent samples T-test was used to compare T^CV^/A^33^ and T^CV^/A^34^ groups. T^CV^, *TYROBP* common variant; T^del^, monoallelic *TYROBP* deletion; A^33^, *APOE* ε3ε3; A^34^, *APOE* ε3ε4.


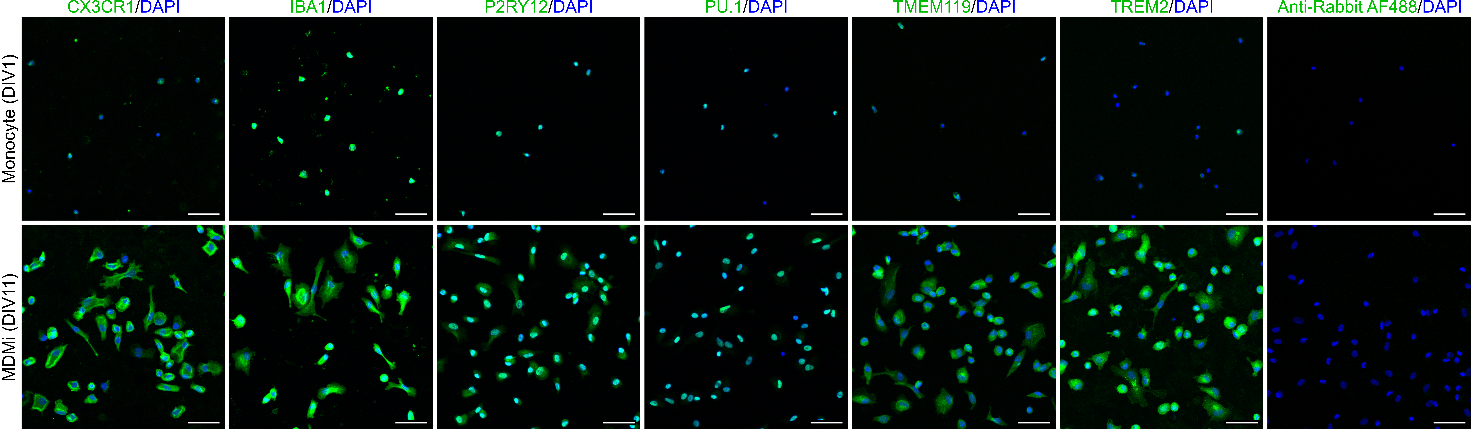


**Fig. S4 Monocyte-derived microglia-like cells (MDMi) express microglial markers CX3CR1, IBA1, P2RY12, PU.1, TMEM119, and TREM2 (green).** Nuclei are labelled with DAPI (blue). Negative controls processed without primary antibodies show no unspecific staining. Scale bars 50 µm. DIV, days in vitro.


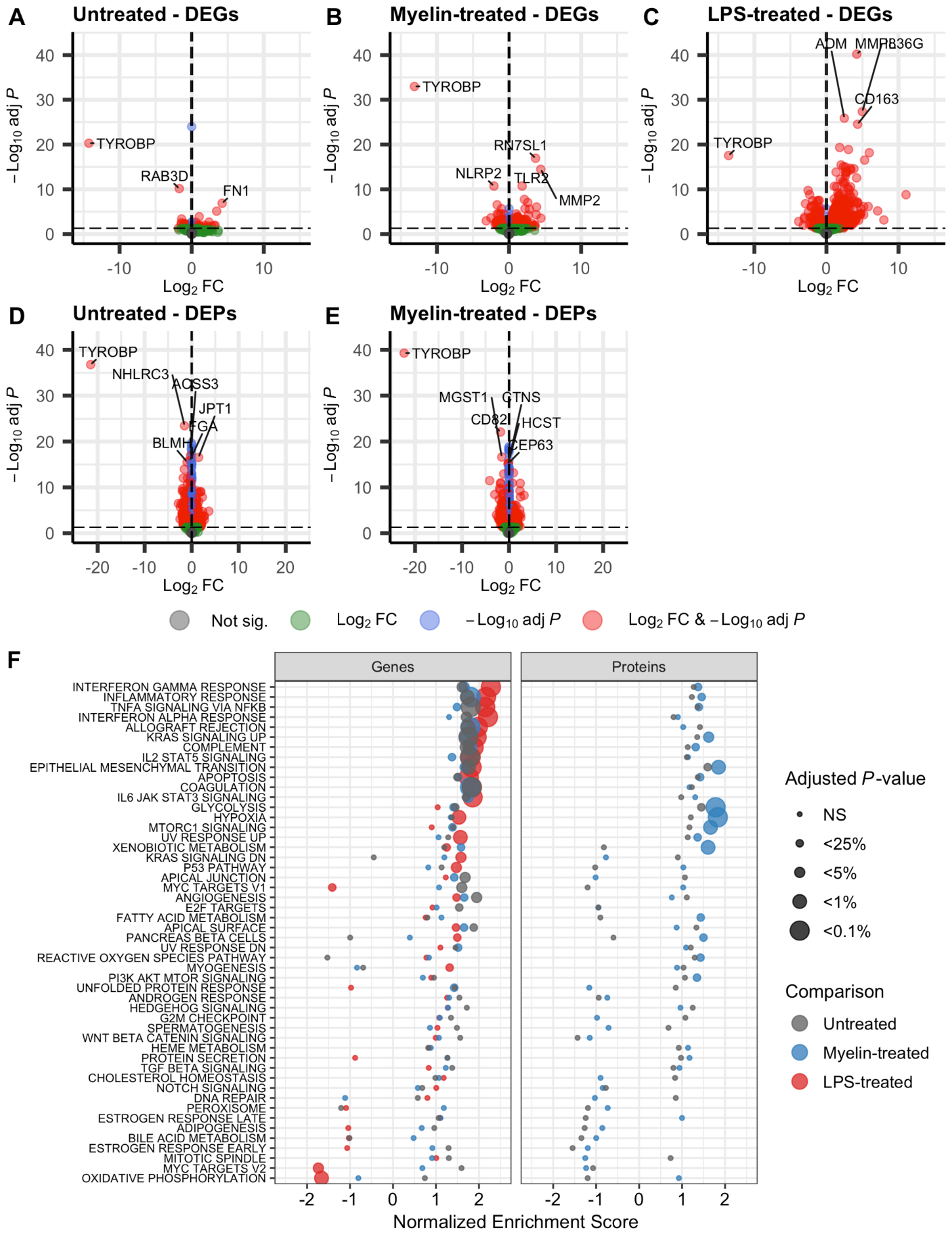


**Fig S5 Increased inflammatory response induced by biallelic *TYROBP* deletion in MDMi cells.** **(A-C)** Differentially expressed genes (DEG) and **(D-E)** proteins (DEP) in NHD patient-derived MDMi cells compared to controls upon untreated (A, D), myelin-treated (B, E) and LPS-treated (C) conditions. **(F)** Pathway enrichment of genes (left panel) and proteins (right panel) differentially expressed in the NHD patient MDMi cells compared to controls. NHD, n=2 (A-E); controls with *TYROBP* common variant, n=12 (A), n=7 (B), n=3 (C-E).


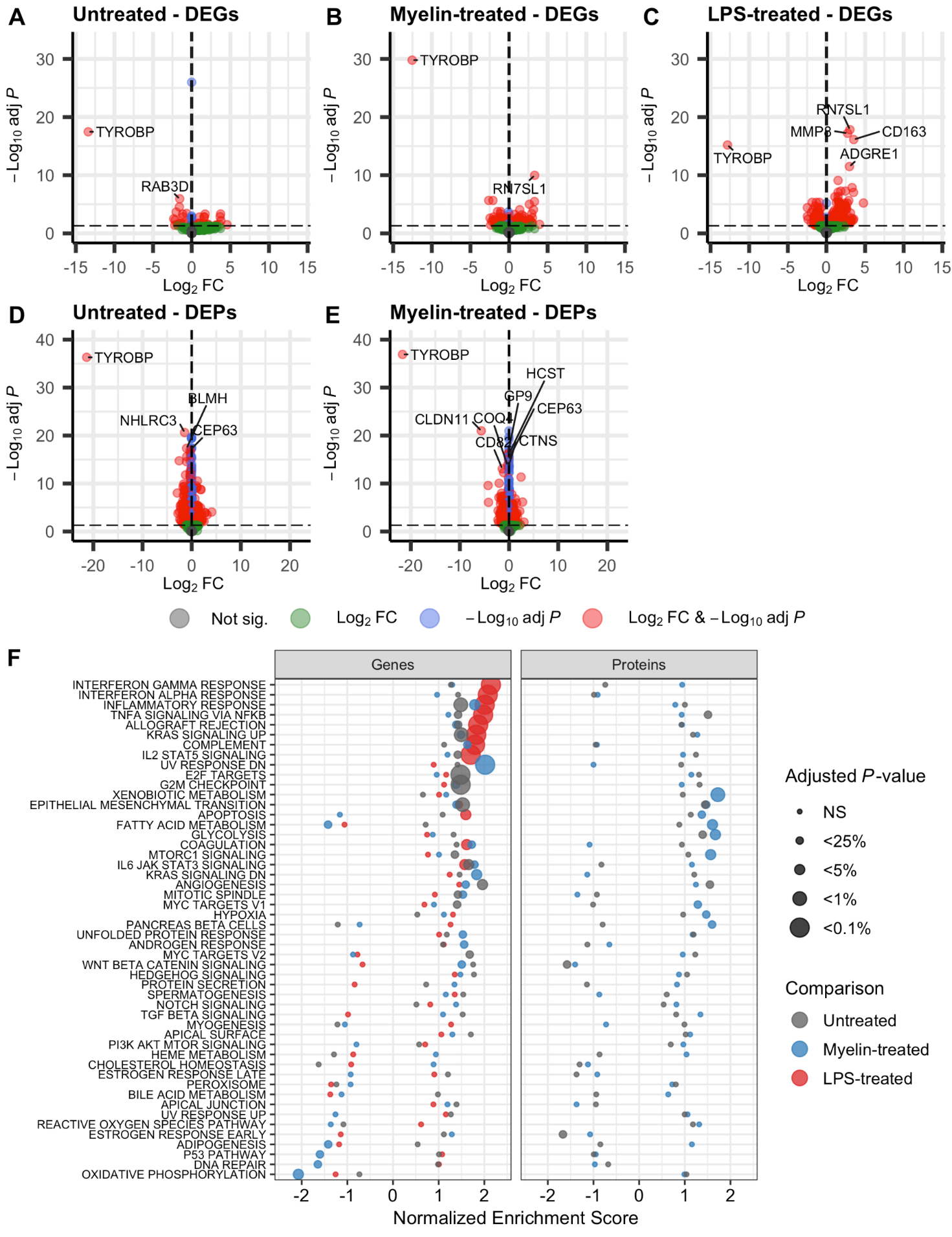


**Fig S6 Altered pathways induced by biallelic *vs*. monoallelic *TYROBP* deletion in MDMi cells.** **(A-C)** Differentially expressed genes (DEG) and **(D-E)** proteins (DEP) in NHD patient-derived MDMi cells compared to monoallelic *TYROBP* deletion carriers upon untreated (A, D), myelin-treated (B, E) and LPS-treated (C) conditions. **(F)** Pathway enrichment of genes (left panel) and proteins (right panel) differentially expressed in the NHD patient MDMi cells compared to monoallelic *TYROBP* deletion carriers. NHD, n=2 (A-E); monoallelic *TYROBP* deletion carrier, n=3 (A-E).


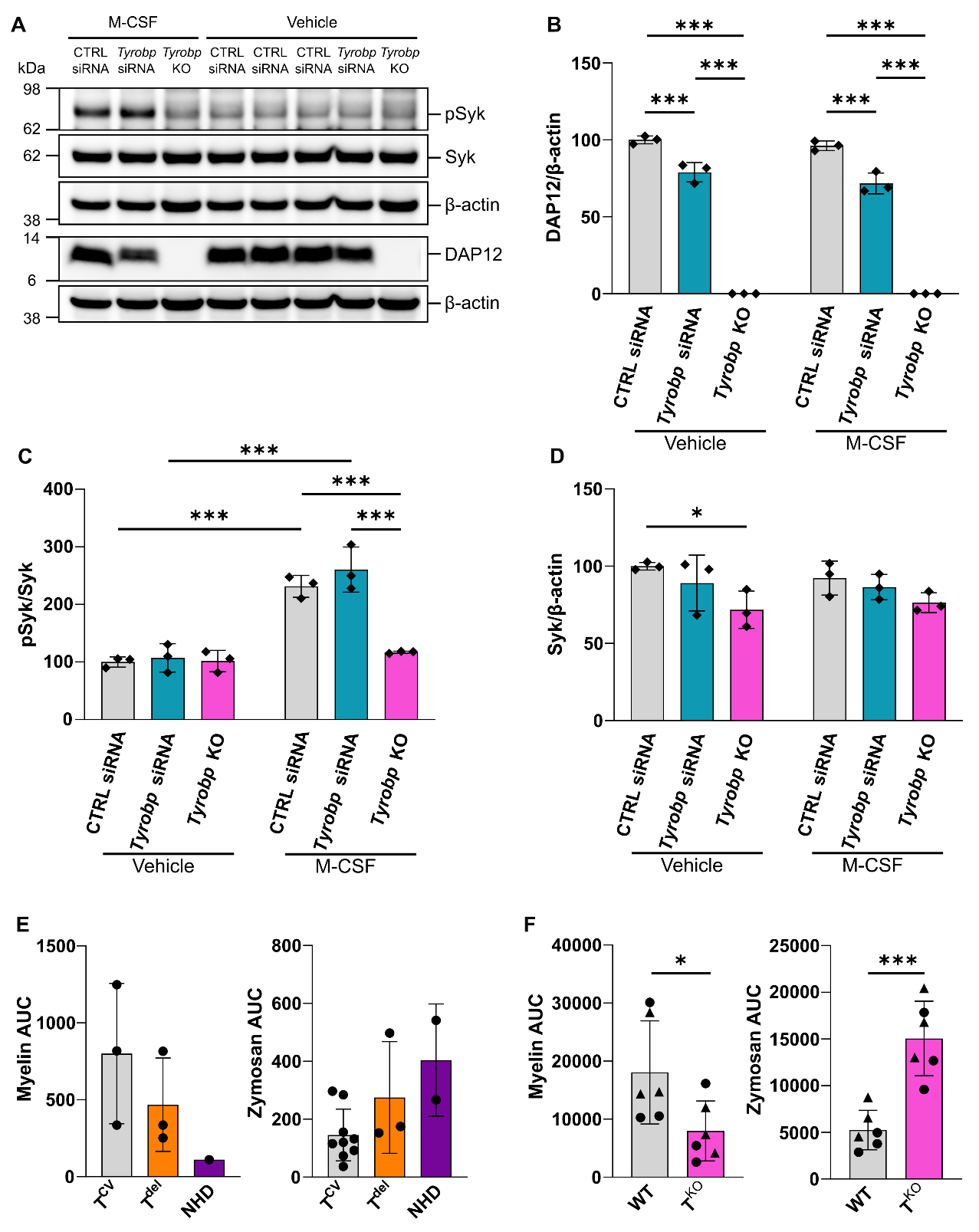


**Fig. S7 M-CSF signalling in DAP12 deficient immortalized BV2 microglial cells. (A)** Western blot of phosphorylated Syk (pSyk), total Syk, and DAP12 in BV2 cells after M-CSF treatment. DAP12 deficiency was obtained by siRNA silencing of *Tyrobp* or by lentiviral CRISPR-Cas9 editing yielding stable *Tyrobp* knockout (KO) cells. Non-targeting control (CTRL) siRNA was used for the control group and *Tyrobp* KO cells. DAP12 was detected from a separate blot. β-actin was used as a loading control. **(B)** Quantification of DAP12 protein normalized to β-actin, **(C)** pSyk normalized to total Syk, and **(D)** total Syk normalized to β-actin. All data in B-D are shown as mean ± SD, n=3, and the values are shown as % of vehicle-treated control siRNA group. Two-way ANOVA with Tukey’s post hoc test. T^KO^, *Tyrobp* KO; WT, wild type. *: *P*<0.05, ***: *P*<0.001.
